# Anti-Aging Effect of Nano-ZnO on Asphalt: Chemo-Rheological Behavior, Molecular Size Evolution of Polymers, and Nanoscale Parameters

**DOI:** 10.3390/polym17202774

**Published:** 2025-10-16

**Authors:** Baifu An, Yang Shen, Jianan Liu, Junmeng Li, Haosen Jing, Shisong Ren

**Affiliations:** 1 School of Resource & Environment and Safety Engineering, Hunan University of Science and Technology, Xiangtan 411201, China; 1010097@hnust.edu.cn (B.A.);; 2 Work Safety Key Lab on Prevention and Control of Gas and Roof Disasters for Southern Coal Mines, Hunan University of Science and Technology, Xiangtan 411201, China; 3 School of Mines, China University of Mining & Technology, Xuzhou 221116, China; lijunmeng1201@163.com; 4 Department of Engineering and Architecture, University of Parma, Parco Area Delle Scienze 181/A, 43121 Parma, Italy; haosen.jing@unipr.it; 5 Sustainable Pavement and Asphalt Research Group, University of Antwerp, 2000 Antwerp, Belgium; Shisong.Ren@uantwerpen.be

**Keywords:** asphalt binder, nano-zinc oxide powder, aging resistance, polymer size evolution, polymer nanocomposites

## Abstract

Asphalt is a widely used polymeric material in pavement engineering. However, it is easily affected by heat and ultraviolet rays, which accelerate its molecular degradation and physicochemical aging, thereby limiting its service life. To improve the anti-aging properties of asphalt, three types of nano-zinc oxide (ZnO)-modified asphalt were prepared. The chemo-rheological behavior, structural evolution of polymeric components, molecular weight distribution, and nanoscale morphology of nano-ZnO-modified asphalt were studied via dynamic shear rheometry (DSR), Fourier transform infrared spectrometry (FTIR), gel permeation chromatography (GPC) and atomic force microscopy (AFM), and the aging resistance of nano-ZnO-modified asphalt was quantitatively analyzed using the rutting factor index, functional group index, molecular size ratio, and nanoscale parameters. The findings indicate that nano-ZnO enhances the high-temperature rheological properties of asphalt and delays the increase in the rutting factor of aged asphalt. Nano-ZnO is dispersed in the asphalt matrix in the form of a physical mixture without inducing new chemical bonds, and can reduce the nanoscale roughness of asphalt. After aging, the nanoscale roughness and the aspect ratio of the bee structure decreased, and the bee structure area increased. According to the changes in the functional group index and the proportions of molecular sizes in the asphalt, it was found that nano-ZnO can significantly improve asphalt’s aging resistance. The results of this study provide insights into the nanoscale modification and structure–property relationships of polymeric asphalt binders, providing a reference for the design and application of functional polymer nanocomposite systems with improved durability.

## 1. Introduction

Asphalt has several benefits that have led to its extensive use in high-grade highways, including excellent driving comfort, reduced noise levels, and rapid construction timelines [1,2]. However, an asphalt mixture can be affected by the environment and load during its production, mixing, transportation, construction, and service use, during which it will continue to age. Aging leads to weakening of the bond between asphalt and aggregate, causing the asphalt to gradually harden and become brittle, which makes it more susceptible to cracking [3,4]. Further, cracks, which are generated as a result of traffic load and temperature, can be penetrated by water and other harmful substances and damage the internal structure of an asphalt pavement, thus affecting its service life [5,6]. Therefore, improving the aging resistance of asphalt materials to delay the aging of asphalt pavements and extend their service life has become a focus of recent research.

Scholars have determined that modifiers can improve the anti-aging properties of asphalt. The authors of [7] used highly reclaimed rubber to prepare asphalt modified with a styrene–butadiene–styrene (SBS) composite and found that it can substantially enhance the aging resistance of asphalt. Liu et al. [8] used the ion exchange method to prepare different antioxidant-intercalated double hydroxides (OLDHs) and added them to SBS-modified asphalt. Their findings demonstrated that incorporating OLDH enhanced resistance to ultraviolet radiation and thermal oxidative aging in SBS-modified asphalt mixtures. Zhang et al. [9] created an organic LDHs/SBS-modified asphalt binder through melt blending. They discovered that the unique layered structure of LDHs can restrict the mobility of asphalt molecular chains and strengthen the interaction between the molecular chains of asphalt and the SBS modifier. This leads to improved resistance against ultraviolet aging. Sha et al. [10] prepared modified asphalt binders using SBR (styrene–butadiene rubber) with EVA (ethylene–vinyl acetate) and conducted thermal oxidative aging experiments. The results showed that the addition of EVA formed a three-dimensional polymer network in the asphalt binder, which enhanced the high-temperature rheological properties, deformation resistance, and fatigue resistance of the asphalt binder. The authors of [11] found that rejuvenators can improve the aging resistance of reclaimed asphalt pavement, with the modification of asphalt using crushed rubber (CR) and recycled polyethylene (PE) significantly improving the aging resistance of the binder blend. In addition, it has been found that well-distributed PE particles can interact with dispersed CR particles to create a network structure that enhances resistance to aging [12].

In addition to the above-mentioned antioxidants and polymer additives, nanomaterial modifiers can change the microstructure of asphalt at the nanoscale. Because nanomaterials are small in size and their surface atoms have many unsaturated dangling bonds, they have small size effects and surface interface effects, so they can scatter ultraviolet light or block thermal oxygen. Therefore, nanomaterial-modified asphalt can improve the aging resistance of asphalt to a certain extent. Some scholars have used carbon nanotube (CNT)- and polyethylene composite-modified asphalt [13], showing that CNT/PE composites exhibit a synergistic effect in enhancing the high-temperature performance of asphalt binders. Additionally, the modified asphalt demonstrates excellent resistance to aging. The authors of [14] also investigated the rheological and microstructural characteristics of montmorillonite (MMT)-modified asphalt, and found that asphalt binder modified with MMT at 4% by weight demonstrates superior resistance to aging.

Nano-TiO_2_- and montmorillonite composite-modified asphalt can significantly delay the conversion of light components to asphaltene during asphalt aging, thereby improving its UV aging resistance [15]. Furthermore, it has been suggested that incorporating a diverse range of nano-quantum dots to alter graphene/titanium dioxide can enhance the UV absorption capability of inorganic nanoparticles [16], thus reducing the UV-induced aging of asphalt pavements. Another study used zinc oxide–silicate composite materials to modify asphalt, and found, through a series of experiments, that it can improve the asphalt’s anti-aging properties [17]. Zhu et al. [18,19] conducted a comprehensive investigation into how zinc oxide/expanded vermiculite influences the physical characteristics, rheological behavior, and chemical composition of asphalt, and confirmed the improved performance of roads comprising asphalt mixtures modified with this compound. Their findings indicate that zinc oxide/expanded vermiculite is capable of significantly reducing the oxidation rate of asphalt over extended aging periods. The results of the abovementioned studies show that nanomaterials can effectively delay the aging of asphalt; however, most studies focus on changes in the physical properties, rheological parameters, and chemical structure of modified asphalt, and the results are mainly qualitative. Moreover, there are relatively few studies that quantitatively analyze the aging resistance of asphalt modified with different nano-ZnO dosages.

In this work, three types of nano-ZnO powder were used to prepare modified asphalt. Their short- and long-term aging effects on various asphalts were simulated through a Rolling Thin-Film Oven Test (RTFOT) and Pressure Aging Vessel (PAV) tests. The rheological properties, chemical composition, molecular weight distribution, and nanoscale morphology of modified asphalt containing different nano-ZnO concentrations were evaluated under diverse aging conditions using the DSR, FTIR, GPC, and AFM techniques, and aging resistance was quantitatively evaluated based on the extent of parameter changes observed before and after the aging process. The core purpose of this study is to compare changes in the aging resistance parameters of nano-ZnO-modified asphalt in different experiments, thereby determining the most suitable experimental and test for evaluating the aging resistance of nano-ZnO-modified asphalt. The results will help us understand the mechanism of nanomaterials’ aging resistance effects on asphalt.

## 2. Materials and Methods

### 2.1. Materials

#### 2.1.1. Asphalt

Virgin asphalt (90#SK Corporation, Seoul, Republic of Korea), was used in this paper, and its basic properties were tested according to specification JTG E20-2011 [20], as shown in Table 1.

#### 2.1.2. Nano-ZnO

**Figure 1:**
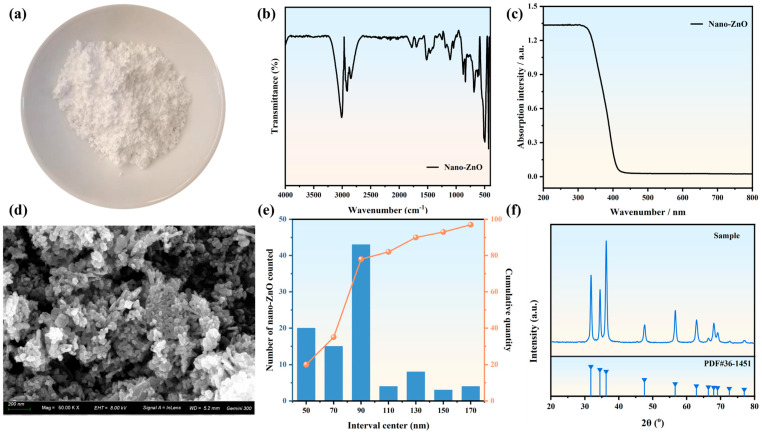
Nano-zinc oxide: (**a**) morphology; (**b**) infrared spectrum; (**c**) UV–visible spectrum; (**d**) microscopic morphology; (**e**) particle size distribution; (**f**) crystal structure.

Nano-ZnO in the form of white powder (as shown in Figure 1a) was obtained from Qinghe Ruineng New Materials Co., LTD., Xingtai, China. Its infrared spectrum and ultraviolet visible spectrum are shown in Figure 1b,c. The infrared spectrum test results for nano-zinc oxide show that it has obvious zinc oxide characteristic peaks at 3150 cm^−1^ to 2950 cm^−1^ and 443 cm^−1^, and the nano-ZnO powder is highly absorbed in the wavelength range of 200 nm to 350 nm [21,22], as shown in Figure 1c. The microstructure of nano-zinc oxide is illustrated in Figure 1d, while Figure 1e displays its particle size distribution. The ZnO sample exhibits a particle size distribution with D50 and D90 of 95 nm and 135 nm, respectively, and a specific surface area of 110 m^2^·g^−1^. The XRD results of nano-ZnO are shown in Figure 1f, which shows that diffraction peaks of certain intensities occur at 31.81°, 34.49°, 36.28°, 47.58°, 56.63°, 62.91°, 66.44°, 67.96°, and 69.14°, and it has the crystal structure of Zincite [23].

### 2.2. Experimental Methods

#### 2.2.1. Preparation Process

After the virgin asphalt was heated to a molten state at 150 °C, samples containing 2%, 3%, and 4% nano-ZnO powder were produced. The modified asphalt samples were sheared at 3500 rpm for 50 min using the ED100 high-speed shearing machine, Claire, Nantong, China, and then stirred at 1500 rpm for 30 min [24] to obtain three types of nano-zinc oxide-modified asphalt (ZnO-2%, ZnO-3% and ZnO-4%). Neither nitrogen nor antioxidants were used in the shear mixing process, and the virgin asphalt was heated and stirred in the same way for comparison.

#### 2.2.2. Asphalt Aging Test

According to the requirements of JTG E20-2011 (T 0609), the asphalt was subjected to short-term aging via a SYO-3061 (85) Thin-Film Oven Test (TFOT), Ruifa Zhongxing, Cangzhou, China. The asphalt was then heated to a fluid state, and 50 g was evenly poured into the bottom of a round metal plate. The asphalt was kept at 163 °C for 5 h to simulate the short-term aging process.

According to the requirements of JTG E20-2011 (T 0630), the long-term aging of asphalt was simulated using a Pressure Aging Vessel (PAV, Changji Geological Instrument Co., LTD., Shanghai, China) on the basis of the short-term aging test. A pressure of 2.1 MPa was applied to the asphalt sample at 100 °C for 20 h, and three parallel samples were used for testing.

#### 2.2.3. Dynamic Shear Rheology Test

The temperature of the three types of asphalt was scanned using an Anton Paar SmartPave 102 dynamic shear rheometer (DSR), Graz, Austria. The temperature range was 46 °C~70 °C, and the test was performed every 6 °C, with a frequency of 10 rad/s and a strain of 0.1% to ensure that all asphalt samples were within the linear viscoelastic range [25]. The rutting factor (G*/sinδ) was calculated, and the asphalt rutting factor aging index was defined by comparing the ratio of the rutting factors before and after aging, as shown in Equation (1) [25]:

(1) $$RFI=\frac{Aged(G^{*}/sin\delta )}{Unaged(G^{*}/sin\delta )}$$

#### 2.2.4. FTIR Test

**Figure 2:**
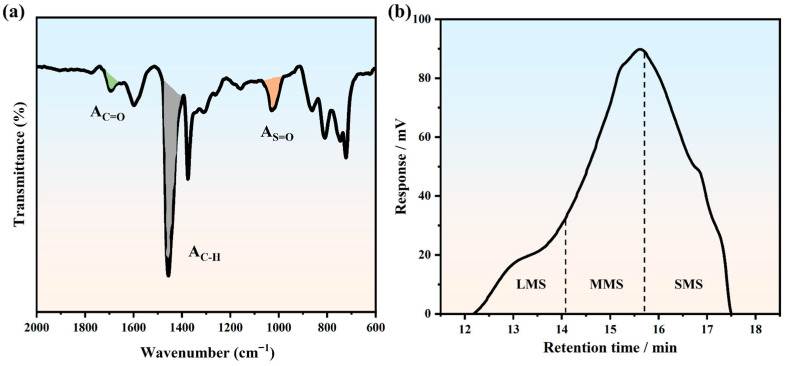
(**a**) Schematic diagram of functional group index calculation. (**b**) Schematic diagram of proportions of different molecular sizes.

FTIR was performed using a Varian 600-IR infrared spectrometer (Agilent, Palo Alto, CA, USA). The binders, after being subjected to various aging conditions, were analyzed within the spectral range of 4000 cm^−1^ to 600 cm^−1^, using a resolution setting of 0.5 cm^−1^ and performing 32 scans. For all the original infrared spectral data, the automatic baseline correction function of OMNIC 9.2 software was used to ensure that the spectral baseline was straight, and at the same time, we eliminated the influence of the background and scattering. To quantitatively analyze the content of carbonyl and sulfoxide groups, the absorption peaks near 1700 cm^−1^ and 1030 cm^−1^ were fitted by peak separation. The peak position, peak height, and half-height width were adjusted using the multi-peak fitting tool in Origin to minimize the fitting residuals, thereby obtaining relatively accurate data. The carbonyl index (*I_C=O_*) and sulfoxide index (*I_S=O_*) (as shown in Figure 2a) characterize the degree of asphalt oxidation during aging, as shown in Equations (2) and (3):

(2) $$I_{C=O}=\frac{A_{C=O}}{A_{C-H}}$$

(3) $$I_{S=O}=\frac{A_{S=O}}{A_{C-H}}$$

where *A_C=O_* represents the area at 1700 cm^−1^; *A_S=O_* represents the area at 1030 cm^−1^; and *A_C-H_* represents the area at 1450 cm^−1^.

#### 2.2.5. GPC Test

The PL-GPC 500 GPC instrument (Agilent Technologies, Church Stretton, UK) was used to study the changes in the proportions of the molecular sizes of asphalt under different aging conditions. A gel chromatograph was used to separate the polymer samples according to the sizes of the molecules. The examination employed polystyrene with a predetermined molecular weight as the reference material, and the molecular weight of the sample was determined by establishing a standard curve and comparing it with the measured data. The resulting solution was then filtered using a 0.45 μm nylon filter before being introduced into the GPC system for analysis [26]. The testing was conducted at a temperature of 30 °C, using tetrahydrofuran (THF) as the washing solvent, with the controlled flow rate set at 1 mL per minute. An asphalt sample characterized the proportion of different molecular sizes in the asphalt according to the elution time and response intensity of molecules of different sizes. In this paper, a GPC chromatogram was produced and divided into 13 fragments according to the elution time. The initial five fragments were categorized as Large Molecular Size (LMS), fragments six to ten were classified as Medium Molecular Size (MMS), and the final four fragments were classified as Small Molecular Size (SMS), as illustrated in Figure 2b [27,28].

#### 2.2.6. AFM Test

**Figure 3:**
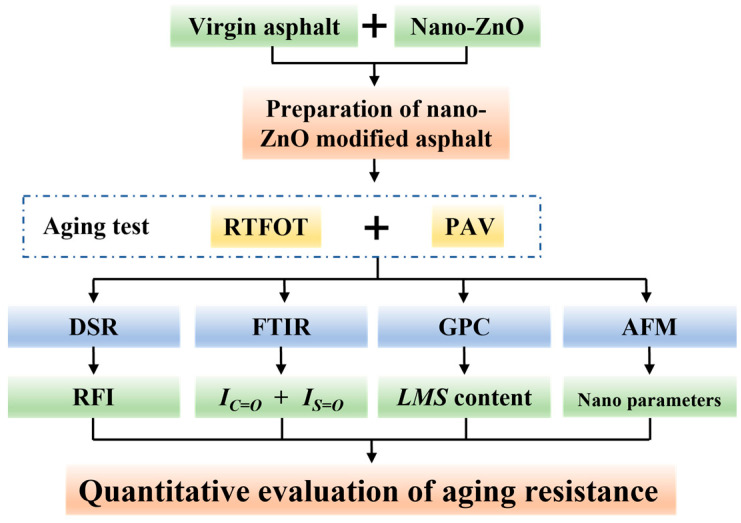
Testing process employed in this study.

The nanomorphologies of the different asphalt samples were tested by Bruker Dimension icon AFM (Billerica, MA, USA). The scanning frequency in the AFM test was 1 Hz, and the surface morphologies of the asphalt matrix and the asphalt modified with 3% nano-ZnO were scanned in tapping mode. In addition, NanoScope Analysis 1.5 was used to perform a statistical analysis on the bee structure of each asphalt sample, with the parameters including image height difference, roughness, average aspect ratio of the bee structure, average area, and others (no fewer than 20 bee structures were counted for each sample) [29]. The testing process employed in this study is shown in Figure 3.

## 3. Results and Discussion

### 3.1. Rheological Properties

#### 3.1.1. Changes in Rheological Parameters of Different Asphalt Samples

**Figure 4:**
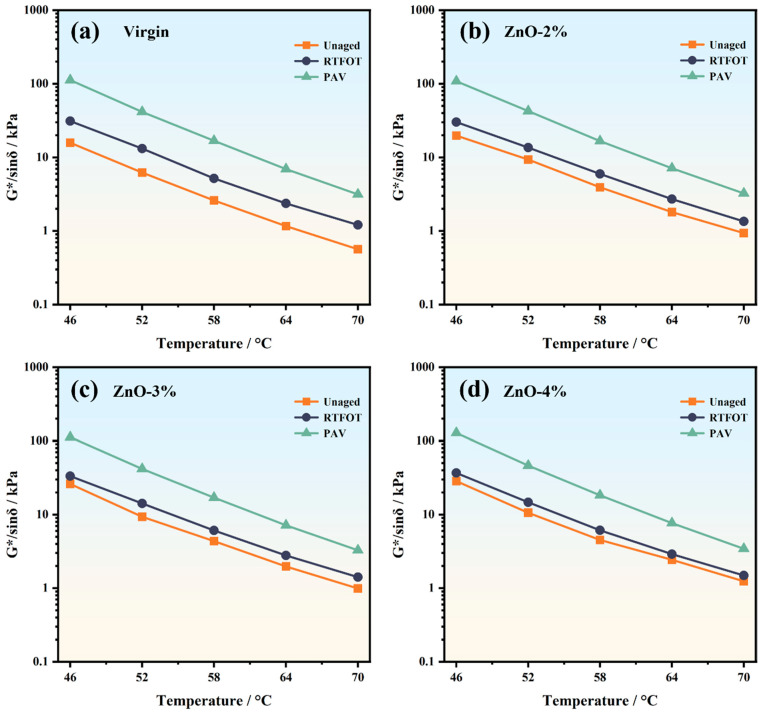
Rutting factors of asphalt under different aging conditions: (**a**) virgin; (**b**) ZnO-2%; (**c**) ZnO-3%; (**d**) ZnO-4%.

By analyzing the G* and δ variations in asphalt, the rutting factor (G*/sinδ) of asphalt under various conditions was determined, as illustrated in Figure 4. G*/sinδ combines the two viscoelastic parameters G* and δ, which reflects the resistance of asphalt to permanent deformation. A larger rutting factor value indicates that the asphalt is more resistant to rutting deformation. The rutting factor of all asphalt is negatively correlated with the test temperature; that is, G*/sinδ is relatively low under higher-temperature conditions. This indicates that at high temperatures, the capacity of asphalt to resist permanent deformation progressively diminishes. The rutting factor of nano-ZnO-modified asphalt is higher under unaged experimental conditions; taking the middle temperature (58 °C) as an example, the G*/sinδ values of ZnO-2%, ZnO-3%, and Zn-O-4% are 3.92 kPa, 4.36 kPa, and 4.51 kPa, respectively, and compared with the rutting factors value of 2.60 kPa in the virgin state, that of the three ZnO-modified asphalt samples increased by 50.70%, 67.57%, and 73.41%, respectively.

Compared with the unaged state, the G*/sinδ of the nano-ZnO-modified asphalt after different aging treatments increases. As the degree of aging increases, the lighter components within asphalt diminish, leading to gradual hardening of the material. After RTFOT, taking the temperature of 58 °C as an example, the rutting factors of virgin asphalt and the three nano-ZnO-modified asphalt samples increased by 2.57 kPa, 2.05 kPa, 1.73 kPa, and 1.60 kPa, respectively. The rutting factor of virgin asphalt in the unaged state is the smallest, but its rutting factor increases the most after RTFOT. This shows that virgin asphalt is greatly affected by short-term aging, while nano-ZnO can reduce changes in the rheological parameters of modified asphalt to a certain extent. After PAV, the rutting factor of virgin asphalt increases to 16.83 kPa, at which point it exceeds the G*/sinδ of the ZnO-2% sample. After PAV, at a temperature of 58 °C, the G*/sinδ of the four types of asphalt increased by 5.47 times, 3.25 times, 3.05 times, and 2.90 times, respectively, compared with the unaged state. Firstly, nano-ZnO particles possess an extremely high specific surface area and surface energy, enabling strong physical adsorption and chemical interactions with light fractions (such as aromatics and saturates) in the asphalt. These interactions facilitate the formation of a more robust nanocomposite structure within the asphalt matrix, which effectively restricts the free movement of asphalt molecular chains at elevated temperatures, thus enhancing the elastic response and resistance to permanent system deformation. These improvements are reflected in the rheological parameters as an increase in G, ultimately leading to a significant enhancement in G/sinδ [30,31].

#### 3.1.2. Evolution of the Aging Index

**Figure 5:**
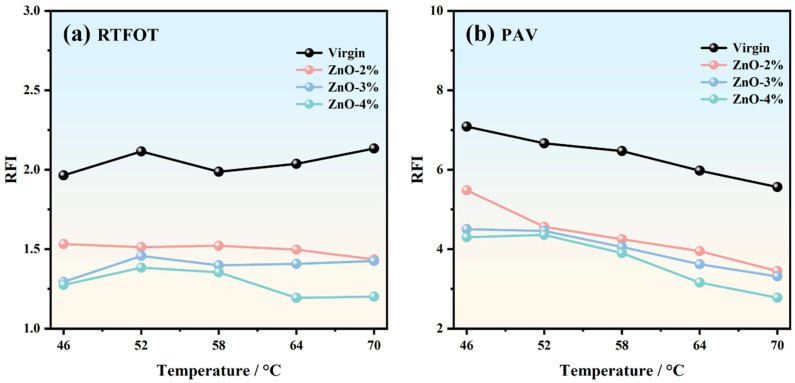
Rutting factor aging indices of different types of asphalt: (**a**) RTFOT; (**b**) PAV.

The short-term aging rutting factor index for the four types of asphalt at various temperatures is presented in Figure 5a. The RFI value of the virgin asphalt is maintained at around 2.0, and this type of asphalt is less affected by temperature, while the RFI values of ZnO-modified asphalt are all lower than 1.6. The RFI value of ZnO-2% is around 1.5, and decreases slightly at higher temperatures. The RFI values of ZnO-3% and ZnO-4% first increase and then decrease with increasing temperature. Taking the temperature of 58 °C as an example, the RFI values of ZnO-2%, ZnO-3%, and Zn-O-4% are reduced by 23.41%, 29.66%, and 31.84%, respectively, compared with the virgin asphalt. The ZnO content is inversely proportional to the RFI value, that is, the asphalt sample with a larger ZnO content has a lower RFI value. The rutting factor indices of the four types of asphalt at different temperatures after PAV are shown in Figure 5b. Unlike in the RTFOT state, there is an obvious relationship between the RFI values of the four asphalt types after PAV and temperature. The RFI value gradually decreases with increasing test temperature, especially at temperatures between 52 °C and 70 °C. That is, under higher-temperature conditions, the RFI values of the four types of asphalt are relatively low. The RFI value of the virgin asphalt after PAV is maintained between 5.5 and 7.1, while the RFI value of the asphalt mixed with ZnO is between 2.7 and 5.5. Taking the temperature of 58 °C as an example, the RFI values of ZnO-2%, ZnO-3%, and Zn-O-4% are reduced by 34.32%, 37.36%, and 39.75%, respectively, compared with virgin asphalt. These results show that ZnO content is closely related to changes in the RFI, and the RFI of the asphalt sample containing ZnO after PAV is more significantly reduced.

### 3.2. FTIR Spectroscopy Analysis

#### 3.2.1. Changes in FTIR of Different Types of Asphalt

**Figure 6:**
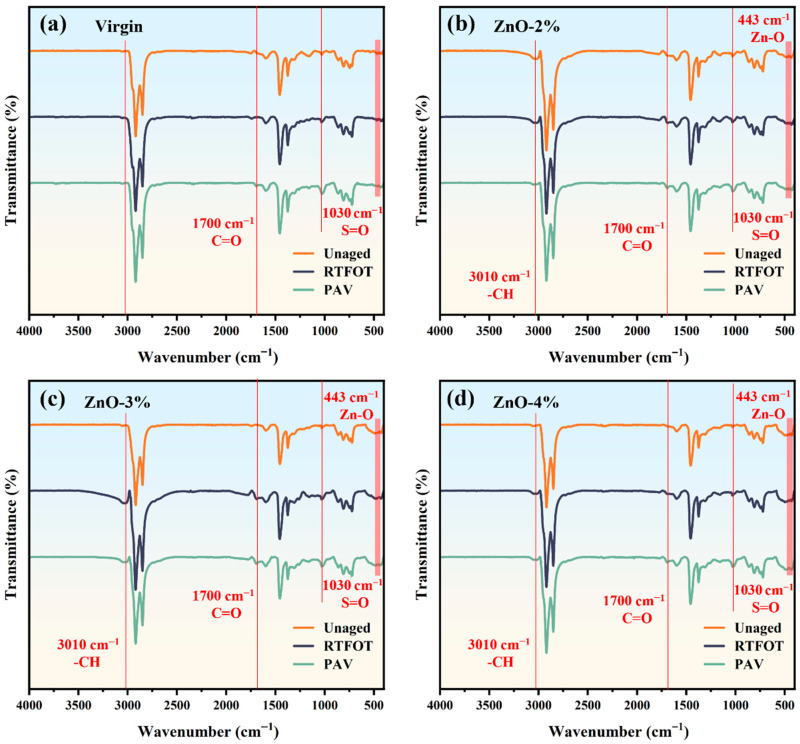
Infrared spectra of asphalt subjected to various aging conditions: (**a**) virgin; (**b**) ZnO-2%; (**c**) ZnO-3%; (**d**) ZnO-4%.

The FTIR spectra for the four types of asphalt under various aging conditions are shown in Figure 6, with Figure 6a showing that the virgin asphalt has C-H bond absorption characteristic peaks of certain intensities at 2920 cm^−1^, 2851 cm^−1^, 1452 cm^−1^, and 1372 cm^−1^. The C-H stretching vibrations of CH_2_ and CH_3_ correspond to 2920 cm^−1^ and 2851 cm^−1^, respectively, while the peak at 1452 cm^−1^ corresponds to the superposition of the -CH_2_- deformation vibration and -CH_3_- asymmetric bending vibration of the methylene group, and the symmetric-bending-vibration absorption peak of -CH_3_ occurs at 1372 cm^−1^. Under the action of aging, the C=O bond of virgin asphalt shows stretching vibration of a certain intensity at 1700 cm^−1^, and the S=O bond shows obvious stretching vibration at 1030 cm^−1^ [32]. Therefore, as the aging effect strengthens, the integrated areas of asphalt at 1700 cm^−1^ and 1030 cm^−1^ gradually increase.

The FTIR spectra of the three types of nano-ZnO-modified asphalt are shown in Figure 6b–d. In addition to the characteristic peaks in the infrared spectrum of the virgin asphalt, the nano-ZnO-modified asphalt presents -CH vibration absorption peaks at 3010 cm^−1^, as well as the characteristic peak of Zn-O at 443 cm^−1^. Figure 1b shows that these two absorption peaks are the characteristic peaks of the nano-ZnO raw material. No new absorption peaks were found except the characteristic peaks of the virgin asphalt and the nano-ZnO raw material. Therefore, there was a physical modification effect between nano-ZnO and asphalt, and no new chemical reactions occurred between them. It is worth noting that during aging, the characteristic peak intensity of the three types of nano-ZnO-modified asphalt at 1700 cm^−1^ and 1030 cm^−1^ gradually increased. The UV scattering ability of ZnO can reduce photochemical oxidation, while ZnO scavenges free radicals through oxygen vacancy sites to enhance the aging resistance of asphalt [33,34]. However, it is difficult to distinguish the specific differences in the four types of asphalt upon being subjected to various aging conditions from the infrared spectra alone.

#### 3.2.2. Functional Group Index Changes

**Figure 7:**
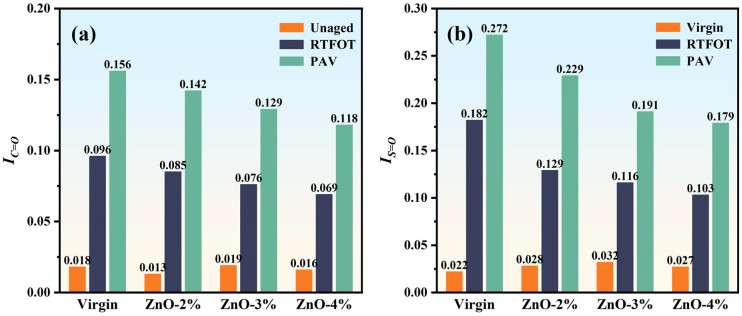
Functional group indices of different types of asphalt: (**a**) *I_C=O_*; (**b**) *I_S=O_*.

As shown in Figure 7, the *I_C=O_* values of the four types of asphalt (virgin, ZnO-2%, ZnO-3% and ZnO-4%) in the unaged state are between 0.013 and 0.019, and their numerical changes have no obvious relationship with the ZnO content. The *I_C=O_* values increase by 0.078, 0.072, 0.057, and 0.053, respectively, under short-term aging via RTFOT, and by 0.138, 0.129, 0.110, and 0.102, respectively, under aging via PAV. We found that the *I_C=O_* value of virgin asphalt increases most significantly (that is, virgin asphalt has the worst aging resistance), while that of nano-ZnO-modified asphalt increases relatively slowly after aging, especially via PAV; however, the *I_C=O_* values of the four types of asphalt after PAV differ by less than 0.04, and the overall difference is not obvious.

The changes in *I_S=O_* and *I_C=O_* are slightly different. The *I_S=O_* value of virgin asphalt after aging increased significantly, from 0.022 to 0.182, and continued to increase to 0.272 after PAV; that is, it increased by 7.27 times and 11.36 times after RTFOT and PAV, respectively. However, the *I_S=O_* value of nano-ZnO-modified asphalt increased relatively slowly, and those of the three types of nano-ZnO-modified asphalt after RTFOT were all below 0.13, while those obtained after PAV were all below 0.23. Based on the changes in the asphalts’ functional group indices following the aging experiments, it is evident that nano-ZnO can substantially enhance asphalt’s resistance to aging.

### 3.3. Molecular-Scale Evolution Analysis

**Figure 8:**
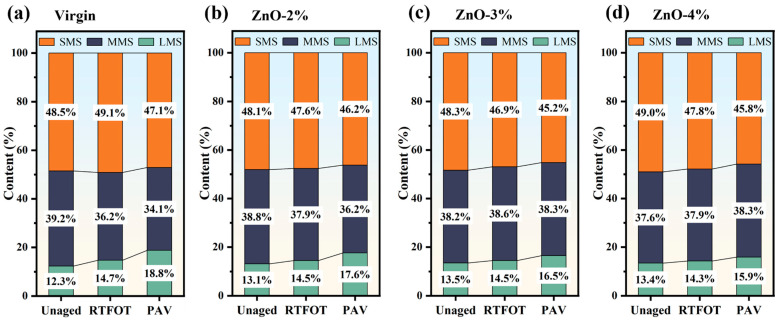
Molecular size ratios of different types of asphalt: (**a**) virgin; (**b**) ZnO-2%; (**c**) ZnO-3%; (**d**) ZnO-4%.

The ratios of molecular sizes for the four types of asphalt under the three aging conditions are presented in Figure 8. During aging, the medium and small molecules are oxidized and polymerized, and their size gradually increases. Specifically, the asphalt’s LMS ratio gradually increases, with that of the virgin asphalt after RTFOT and PAV increasing to 14.7% and 18.8%, respectively, 2.4% and 6.5% higher than in the unaged state. The initial LMSs of the three types of nano-ZnO-modified asphalt are larger (13.1%, 13.5%, and 13.4%, respectively) than that of the virgin asphalt (12.3%). However, the increment in the LMS ratios of the three types of nano-ZnO-modified asphalt after aging is relatively low. After RTFOT, their LMS ratios are between 14.3% and 14.5%, and after PAV, they are 17.6%, 16.5%, and 15.9%, respectively. The above results indicate that the LMS of nano-ZnO-modified asphalt after PAV is lower than that of virgin asphalt, and the LMS growth of asphalt with a larger ZnO content is smaller. Therefore, from the perspective of changes in the proportions of molecular sizes, the addition of nano-ZnO can improve the anti-aging performance of asphalt.

### 3.4. Atomic Force Microscopy Analysis

#### 3.4.1. Nanomorphology

**Figure 9:**
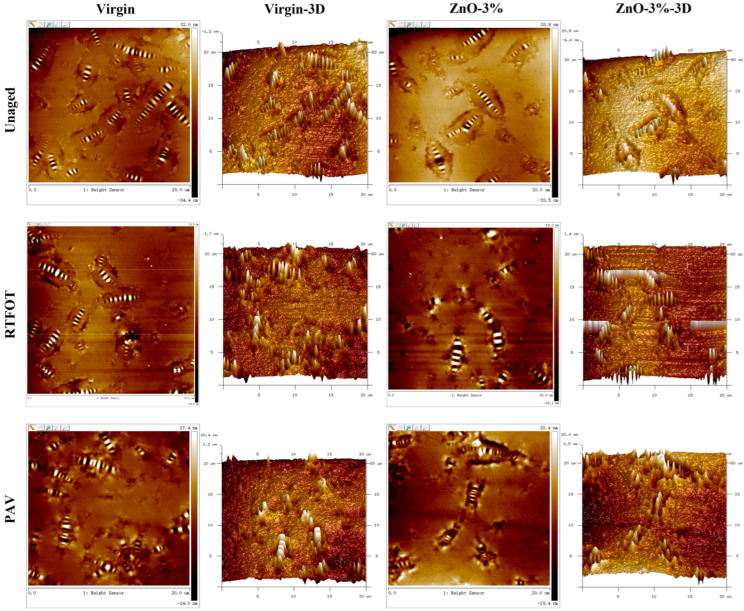
Nanomorphologies of asphalt samples (virgin and ZnO-3%) under different aging conditions (unaged, RTFOT, and PAV).

The ZnO-3% sample was compared with the virgin sample, and their nanomorphologies upon being subjected to various aging conditions are shown in Figure 9. A bee structure with alternating “light and dark” can be seen in both asphalt samples. The 3D morphology better reflects the height relationship between the “light and dark” phases of the asphalt bee structure, where the “light” phase is the peak, and the “dark” phase is the trough. A black dot-like substance can be found in the two-dimensional nanomorphology of ZnO-2%, which is related to the incorporation of nano-ZnO. In the unaged state, the bee structure in virgin asphalt is relatively slender, that is, the aspect ratio is relatively large. The bee structures in ZnO-3% are of different sizes, and there are fewer of them than in the virgin asphalt. After RTFOT, the nanomorphologies of the two types of asphalt are more regular than those in the unaged state (according to the 3D image), and the quantity of bee structures is slightly decreased. The nanomorphologies of the two types of asphalt after PAV are similar to those in the RTFOT state, and the morphology diagram shows no obvious difference between the asphalt samples in the two aging states.

#### 3.4.2. Nanomorphological Parameters

**Figure 10:**
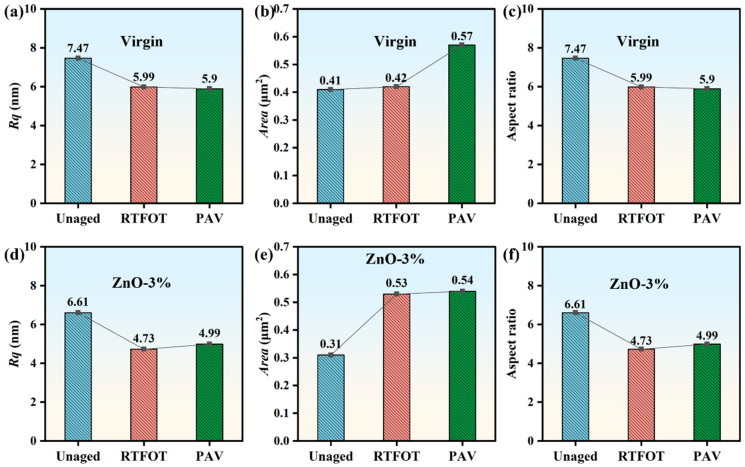
(**a**–**f**) Changes in nano-parameters of asphalt (virgin and ZnO-3%) under different aging conditions (unaged, RTFOT, and PAV).

The bee structure nanomorphological parameters of the two types of asphalt under the three aging conditions were statistically analyzed, including nano-scale roughness (Rq), bee structure area (Area), and bee structure aspect ratio (As-r). Among them, the bee structure area and aspect ratio were measured for no fewer than 20 bee structures, and their average values were calculated; the results are shown in Figure 10, from which it is evident that the nano-scale roughness of the two types of asphalt after aging showed a downward trend. The Rq of virgin and ZnO-3% asphalt after RTFOT decreased by 19.81% and 28.44%, respectively. However, their Rq values after RTFOT and PAV were not considerably different. From the perspective of bee structure area, that of virgin asphalt did not change much after RTFOT, but it increased significantly after PAV; meanwhile, that of ZnO-3% increased by 70.97% after RTFOT, but it did not change much after PAV. The bee structure aspect ratios of both types of asphalt decreased after aging, but the difference in ratios between the RTFOT and PAV states was not significant. In summary, following the aging process, both the Rq and bee structure aspect ratio of virgin and nano-ZnO-modified asphalt decreased, while the bee structure area increased.

## 4. Conclusions

This study investigated the rheological properties, infrared spectra, and nanomorphology of nano-ZnO-modified asphalt. The changes in rheological parameters, functional group indices, and nanomorphological parameters of different modified asphalts were measured and statistically analyzed to evaluate the effect of nano-ZnO on the anti-aging properties of asphalt. The conclusions are as follows:

(1) Nano-ZnO effectively delays the growth rate of the rutting factor during the aging process. Due to its high stiffness and reinforcement effect, the incorporation of nano-ZnO increases the rutting factor of asphalt in the unaged state. Taking the temperature of 58 °C as an example, after RTFOT, the RFI values of ZnO-2%, ZnO-3%, and Zn-O-4% were reduced by 23.41%, 29.66%, and 31.84%, respectively, compared with the virgin asphalt; meanwhile, after PAV, their RFI values were reduced by 34.32%, 37.36%, and 39.75%, respectively.

(2) The modification of asphalt with nano-ZnO occurs through physical blending without new chemical bond formation. The introduction of nano-ZnO slows the increase in oxidative functional groups (*I_C=O_* and *I_S=O_*) during aging. The I_S=O_ value of the asphalt matrix after RTFOT was 0.182, and continued to increase to 0.272 after PAV; meanwhile, the I_S=O_ values of the three types of nano-ZnO-modified asphalt after RTFOT were all below 0.13, and those after PAV were all below 0.23.

(3) The large-molecular-size (LMS) fraction of asphalt gradually increased upon aging, reflecting polymer chain growth and structural evolution. The LMS ratio of nano-ZnO-modified asphalt in the unaged state was higher than that of virgin asphalt. After RTFOT, the LMS ratios of the three types of nano-ZnO-modified asphalt and virgin asphalt were similar, while after PAV, they were lower than that of the virgin asphalt, and a higher ZnO content was associated with a smaller LMS growth rate.

(4) All asphalts exhibited the characteristic “bee structure” morphology with alternating light and dark regions. The addition of nano-ZnO led to localized agglomeration, manifesting as black spot-like domains in the matrix. The nano-scale roughness (Rq) and aspect ratio of the bee structure of virgin and nano-ZnO-modified asphalt after aging were reduced, and the bee structure area increased.

## 5. Challenges and Future Studies

The correlations between AFM image data on the changes in nano-ZnO-modified asphalt and other properties of asphalt have not yet been established. Future research needs to further explore the changes in the nanomorphology of asphalt under different aging states and establish the correlations among multiple test results. Therefore, in the future, we aim to study the correlations between the results of molecular dynamics simulations and laboratory experiments.

## Figures and Tables

**Table 1:** Properties of virgin asphalt.

Parameter	Asphalt	Test Method
Penetration (25 °C, 100 g, 5 s; 0.1 mm)	91	T0604
Ductility (15 °C, 5 cm/min; cm)	>100	T0605
Softening point (°C)	46.5	T0606
Density (g/cm^3^)	1.02	T0603
Solubility (%)	99.7	T0607
Flash point (°C)	296	T0611

## Data Availability

All data is contained within the article.
